# A Novel Role for CAMKK1 in the Regulation of the Mesenchymal Stem Cell Secretome

**DOI:** 10.1002/sctm.17-0046

**Published:** 2017-07-08

**Authors:** Feng Dong, Shyam Patnaik, Zhong‐Hui Duan, Matthew Kiedrowski, Marc S. Penn, Maritza E. Mayorga

**Affiliations:** ^1^ Integrative Medical Sciences Northeast Ohio Medical University Rootstown Ohio USA; ^2^ Computer Science University of Akron Akron Ohio USA; ^3^ Cardiovascular Department Summa Cardiovascular Institute, Summa Health System Akron Ohio USA

**Keywords:** Mesenchymal stem cells, Secretome, Cardiac disease, Cardiac regeneration, Calcium/calmodulin‐dependent protein kinase kinase‐1

## Abstract

Transplantation of adult stem cells into myocardial tissue after acute myocardial infarction (AMI), has been shown to improve tissue recovery and prevent progression to ischemic cardiomyopathy. Studies suggest that the effects of mesenchymal stem cells (MSC) are due to paracrine factors released by MSC, as the benefits of MSC can be achieved through delivery of conditioned media (CM) alone. We previously demonstrated that downregulation of Dab2 enhances MSC cardiac protein expression and improves cardiac function after AMI following MSC engraftment. In order to define the molecular mechanisms that regulate MSC secretome, we analyzed gene arrays in MSC following downregulation of Dab2 via TGFβ1 pretreatment or transfection with Dab2:siRNA or miR‐145. We identified 23 genes whose expressions were significantly changed in all three conditions. Among these genes, we have initially focused our validation and functional work on calcium/calmodulin‐dependent protein kinase kinase‐1 (CAMKK1). We quantified the effects of CAMKK1 overexpression in MSC following injection of CM after AMI. Injections of CM from MSC with CAMKK1 over‐expression correlated with an increase in vascular density (CAMKK1 CM: 2,794.95 ± 44.2 versus Control: 1,290.69 ± 2.8 vessels/mm^2^) and decreased scar formation (CAMKK1 CM 50% ± 3.2% versus Control: 28% ± 1.4%), as well as improved cardiac function. Direct overexpression of CAMKK1 in infarcted tissue using a CAMKK1‐encoding plasmid significantly improved ejection fraction (CAMKK1: 83.2% ± 5.4% versus saline: 51.7% ± 5.8%. Baseline: 91.3% ± 4.3%) and decreased infarct size after AMI. Our data identify a novel role for CAMKK1 as regulator of the MSC secretome and demonstrate that direct overexpression of CAMKK1 in infarcted cardiac tissue, results in therapeutic beneficial effects. Stem Cells Translational Medicine *2017;6:1759–1766*

**Significance Statement**

Stem cells therapy is a promising approach for the treatment of cardiovascular diseases. However, molecular pathways, mechanisms of action, and proper delivery systems are still to be optimized for clinical development. In this study, we analyzed the effect of mesenchymal stem cell secretome on cardiac recovery after acute myocardial infarction. We observed that overexpression of the protein kinase calcium/calmodulin‐dependent protein kinase kinase‐1 (CAMKK1) in either mesenchymal stem cells or upon direct injection of its encoding DNA into infarcted tissue results in improved cardiac function and increased vasculogenesis. Thus CAMKK1 may be suitable as a therapeutic target for cardiac disease.

## Introduction

The beneficial effects of stem cell therapy on the prevention of myocardial injury and restoration of cardiac function after acute myocardial infarction (AMI), has been extensively documented in animal models of cell stem cell transplantation 1, 2. Interestingly, the low survival rate of stem cells in myocardial tissue combined with the limited evidence for differentiation or regeneration of cardiac myocytes by transplanted cells 2, 3 has led many to conclude that the benefits associated with adult stem cell therapy are mainly due to paracrine effects 4, 5. Molecules within the mesenchymal stem cells (MSC) secretome may have suppression effects on the local immune system 6, enhance stem cell homing 7, 8, enhance angiogenesis 9, inhibit apoptosis, and decrease scar formation 10.

In previous studies by our group, we demonstrated that the ability of transplanted MSC to restore cardiac function after AMI was significantly enhanced if the MSC were pretreated with the growth factor TGFβ1 9, 11. This effect was mediated through the modulation of intracellular signaling pathways involving upregulation of miR‐145, and subsequent downregulation of the TGFβ1 receptor adaptor protein with tumor suppressor activity, Dab2. We further observed that downregulation of Dab2 led to greater cardiac protein expression in MSC, which correlated with a significant improvement in cardiac function after AMI 9, 11. In our experimentation, we observed that Dab2 modulates mesenchymal stem cells (MSC) genetic and functional responses potentially involving changes in the pool of MSC secreted proteins and factors, which in turn positively activate damaged tissue as well as surrounding healthy cells, ultimately resulting in improved cardiac function.

In the present study, we demonstrate that the effects of Dab2 downregulation are mediated through changes in the MSC secretome. We then dissect the signaling pathways regulating production of the MSC secretome and identify a novel role for calcium/calmodulin‐dependent protein kinase kinase‐1 (CAMKK1) as a key regulator of the MSC secretome and as a potential strategy for cell‐free induction of MSC‐like repair in AMI.

## Materials and Methods

### Animals

Eight‐week‐old male Lewis rats were maintained under standard conditions in the animal facility at Northeast Ohio Medical University. All animal procedures were approved by the Institutional Animal Care and Use Committee.

### MSC Preparation

MSC were isolated from rat bone marrow of adult Lewis rats. The cells were isolated by flushing the femurs with 0.6 ml Dulbecco's modified Eagle's medium (DMEM) (Gibco, Invitrogen, http://www.invitrogen.com, Carlsbad, CA, https://www.fishersci.com). Clumps of bone marrow were gently minced with an 18‐gauge needle and then centrifuged for 5 minutes at 260 g and washed with two changes of phosphate‐buffered saline (Invitrogen). The washed cells were then resuspended and plated in DMEM‐Low Glucose (Gibco, Invitrogen) with 10% fetal bovine serum (FBS) (Gibco, Invitrogen). The cells were incubated at 37°C, and nonadherent cells were removed by replacing the medium after 3 days. When cultures became 70% confluent, adherent cells were detached following incubation with 0.05% trypsin and 2 mM EDTA (Invitrogen) for 5 minutes. MSC cultures were then depleted of CD45+ and CD34+ cells simultaneously by negative selection using 1 μl each of primary phycoerythrin‐conjugated mouse anti‐rat CD45 (BD Biosciences, http://www.bdbiosciences.com, San Diego, CA) and CD34 antibodies (Santa Cruz Biotechnology, https://www.scbt.com, Santa Cruz, CA) per 10 × 6 cells using the EasySep PE selection kit (Stem Cell Technologies, Vancouver, Canada, https://www.stemcell.com/). Finally, Icam‐positive cells were selected by cell sorting.

### MSC and MSC‐Conditioned Medium

In preparation for transplantation, MSC transfection was performed by Neon Transfection system electroporation. We transfected 2 × 10^6^ cells with siRNA:CAMKK1 or cDNA:CAMKK1, with transfection efficiency of 55%–80%. Conditioned medium was generated as follows: After eleven passages, 90% confluency was noted in both the control and experimental groups. Conditioned medium was generated by feeding cells transfected with cRNA:CAMKK1 with serum‐free DMEM and incubated for 48 hours. Control medium was generated by using only cells without treatment. The medium was collected and the cells were counted for normalization purposes: For each animal, we used medium generated by 2 × 10^6^ cells. After removing the cellular debris, supernatants were transferred to 12‐ml tubes. A total of 600 μl of conditioned or control medium was injected in five different sites at the infarct border zone immediately after the left anterior descending (LAD) ligation.

### LAD Ligation to Induce MI

Myocardial infarction was induced in rats using a surgical microscope (LeicaMicrosystems M500) as previously described 9, 10, 11, 12. Briefly, animals were anaesthetized with intraperitoneal ketamine and xylazine, intubated and ventilated with room air at 80 breaths/minutes using a pressure‐cycled rodent ventilator (RSP1002; Kent Scientific Corporation). Sternotomy was performed and the proximal LAD was ligated with 7–0 prolene. Observed blanching and dysfunction of the anterior wall verified LAD ligation. After LAD ligation, the animals were evaluated with echocardiography and euthanized at different time points for organ harvest (heart) and staining.

### Echocardiographic Assessment of Heart Function

Two‐dimension echocardiography was performed with a 15‐MHz linear array transducer interfaced with a Sequoia C256 (Acuson) as preciously described 10, 11 Briefly, baseline and left ventricular (LV) dimensions were quantified pre‐LAD ligation as well as 2 and 8 weeks after LAD ligation, LV dimensions were quantified by digitally recorded 2D clips and M‐mode images from the mid‐LV just below the papillary muscles to allow for consistent measurements from the same anatomical location in different rats. Ejection fraction (EF), fractional shortening, diastolic thicknesses of the LV posterior wall, systolic thicknesses of the LV posterior wall, diastolic LV internal dimensions, and systolic LV internal dimensions were measured. Echocardiographic measurements were performed and analyzed by investigators who were blinded to the treatment and identity of the rat.

### Myocardial Infarct Size Measurements

We measured the area at risk and infarct zone as described previously 8, 9, 11, 12. Briefly, the rat hearts were harvested and perfusion fixed with Hystochoice at physiological pressures 2, 4, and 8 weeks post‐MI. Fixed hearts were embedded in paraffin and serially cut at 4 μm from the apex to the level just below the coronary artery ligation site. Alternating sections were stained with Masson trichrome. The infarcted area was measured by planimetry with use of computer‐assisted image analysis software Image‐Pro Plus (Media Cybernetics). The volume of myocardium at risk and infarcted myocardium was calculated using the following equation: % infarct size = epicardial infarct length/epicardial LV circumferences × 100. Samples were blinded and randomized prior to analysis.

### Vessel Density Measurement

Two, four, six, and eight weeks post‐AMI, tissue was prepared as described above for immunostaining. Sections were stained with fluorescein‐conjugated isolectin (Vector Laboratories), which stains endothelium, and rhodamine‐conjugated wheat germ agglutinin (Vector Laboratories), which labels myocyte membranes as previously described. Five to ten randomly selected fields for each sample were imaged at ×60 by use of confocal microscopy and vessel density within the infarct border zone was measured with ImageJ.

### Gene Array Analysis

The labeled cDNA samples were hybridized to the Illumina Rat Ref‐12 expression BeadChip, which was scanned using Illumina Beadstation GX (Illumina, San Diego, CA). Each BeadChip contains 22,523 probes, which were selected from the National Center for Biotechnology Information (NCBI) Reference Sequence (RefSeq) database. The microarray image was analyzed and intensity data were normalized using Illumina Beadstudio software (Illumina, San Diego, CA). To select differentially expressed genes, a Welch two‐sample *t* test was performed. The results were combined with fold change and detection *p* values to identify differentially expressed genes. Specifically, the selection criterions include the sum of the detection *p* values for the three repeats being less than or equal to .1, average *p* values less than or equal to .05, and fold change between control and treated samples greater than or equal to 1.5. In addition, set operations were performed to identify commonly deregulated molecules. Following the identification of differentially expressing genes, the dataset containing these genes and the corresponding expression values were uploaded into the Ingenuity Systems Pathway Analysis (IPA) software (Ingenuity Systems, Redwood City, CA). TGFβ, miR145, and Dab‐2 as well as these differentially expressed genes were marked as focus molecules in IPA. The focus molecules served as seeds and their relationships with other molecules in the Ingenuity Knowledge Base were identified and presented in a set of networks (directed graphs) in which the biological relationship between two molecules (nodes) is represented as a directed edge. In addition, functional and canonical pathway analyses were carried out using IPA. The focus molecules and their closely related genes were analyzed and over‐represented functional groups and canonical pathways were identified. Significance of association between these genes and a functional group or a canonical pathway was measured using the *p* value obtained using Fisher's exact test determining the probability that the association between the focus molecules and the group/pathway is explained by chance alone. A cutoff threshold of .05 was used in this study.

### Statistics

Data are represented as mean ± SEM. Comparisons between two groups were made with a two‐tailed Student *t* test with significance defined as *p* < .05. Comparisons among multiple groups were made with analysis of variation (ANOVA).

## Results

### Effects of Regulating MSC Secretome in Cardiac Repair

**Figure 1:**
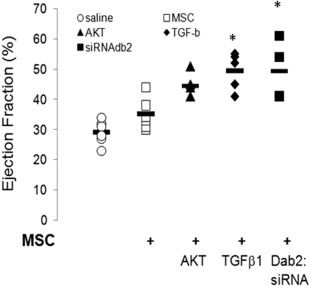
MSC conditioned media (CM) improves cardiac function after acute myocardial infarction. Ejection fraction as a measure of cardiac function, was measured 2 weeks after left anterior descending ligation in hearts injected with concentrated CM from the indicated treatments. TGFβ1 treatment and transfection with Dab2 siRNA, conditions where Dab2 expression is decreased, showed a significant improvement in cardiac function compared with saline injections and CM from control, untreated MSC. Data represent individual animals. *n* = 6–8 per group. *, *p* < .05 versus MSC/control group. Abbreviations: AKT, protein kinase B; MSC, mesenchymal stem cells.

We determined whether the benefits of MSC with downregulation of Dab2 required cell transplantation and engraftment. We generated concentrated conditioned media (CM) from control MSC (transfected with scramble siRNA), TGFβ1 (5 ng/ml) treated MSC and MSC transfected with Dab2:siRNA or Dab2:cDNA. CM from adenosine tyrisone kinase protein (Akt) overexpressing MSC was included as positive control 11, 12, 13. After LAD ligation, rats were randomly divided into five groups and received 600 μl of CM in five divided doses around the infarct border zone within 30 minutes of LAD ligation. Repeat echocardiograms were obtained at 2 weeks after AMI and were compared to baseline. As seen in Figure 1, injection of MSC CM improved LV function (*p* < .02) at 2 weeks after treatment compared with saline control in our rat myocardial infarct model. The benefit of MSC CM was significantly enhanced by downregulation of Dab2 in MSC (*p* < .03), treatment with TGFβ1 (*p* < .005) or upregulation of Akt (*p* < .02). These findings are similar to what we observed with the injection of MSC treated with Dab2:siRNA 9, 11, 12, suggesting that alterations in paracrine factor expression is an important mechanism associated with the effects of Dab2:siRNA treated MSC. In an attempt at identifying potential paracrine factors participating in beneficial cardiac effects observed in our experiments, we measured a series of known secreted proteins in CM, previously subject to Dab2 modulating treatments. The levels of FGF‐2, PDGF‐β, IGF‐1, SDF‐1, and SFRP‐2 in the CM were unchanged in all conditions compared with untreated MSC (data not shown).

### Identification of Pathways Potentially Involved in MSC Secretome Mediated Cardiac Repair

**Figure 2:**
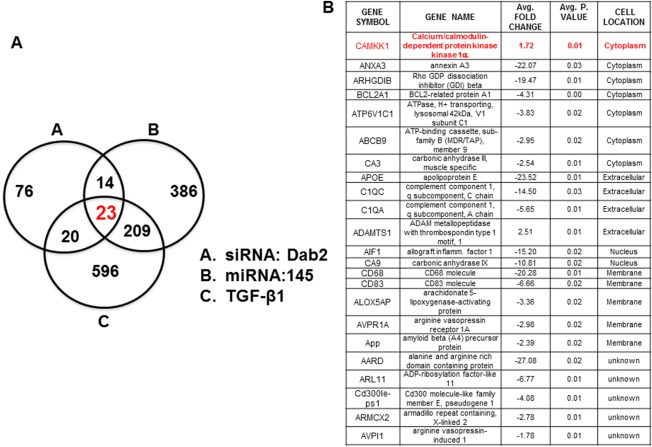
Gene expression analysis of the effect of Dab2 downregulation in mesenchymal stem cells (MSC). Gene array analysis in cells with downregulated Dab2 expression (siRNA:Dab2, miRNA145, TGFβ1), resulted in identification of 23 proteins being significantly affected by these treatments. Upon experimental validation of these genes in MSC, CAMKK1 was selected for further analysis. **(A):** Three circle analysis of gene array results. **(B):** Genes showing significant fold changes in the three experimental conditions.

In order to identify genes potentially involved in the effect of Dab2 deficiency on secreted proteins inducing cardiac beneficial effects, we quantified gene expression in response to three treatments that downregulate MSC Dab2 expression, specifically: Dab‐2:siRNA, miR‐145 upregulation and pretreatment with TGFβ1 (Fig. 2). The focus molecules and their closely related genes were analyzed and overrepresented functional groups and canonical pathways were identified. A cutoff threshold of 0.05 was used in this study. The expression of 23 genes was consistently and significantly changed by all three treatments compared with control (Fig. 2A, 2B). Some of these genes belong to known cardiac cell signaling pathways or structural components (e.g., Anexin A3, Apolipoprotein E and carbonic anhydrase III muscle‐specific). These results also confirm our previous reports on the role of the TGFβ1/Dab2 signaling pathway in regulating cardiac protein expression in MSC. Upon extensive validation of array results, in terms of verifying gene expression variation of individual proteins showing higher fold changes, we focused our attention on CAMKK1. We chose to focus on CAMKK1 due to that fact that it was overexpressed, is a kinase that could lead to down‐stream signaling, and it is a novel protein to the field of regenerative medicine.

### CAMKK1 Expression Is Modulated by Dab2 in MSCs

**Figure 3:**
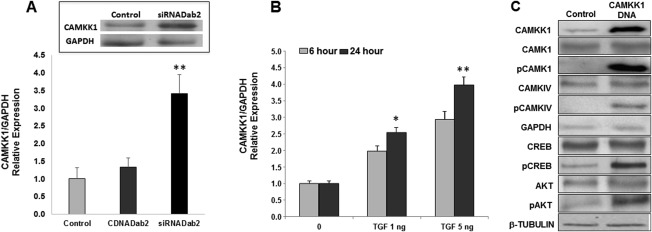
CAMKK1 expression is regulated by Dab2 in mesenchymal stem cells (MSC). Downregulation of Dab2 in MSC by means of siRNA **(A)** or TGFβ1 treatment **(B)**, resulted in increased expression of CAMKK1 as measured by qPCR (A, bar graph) and western blot (A, inset). Results are presented as relative expression of the indicated gene versus GAPDH and are mean ± SEM of two to four independent experiments. Data are normalized to control levels (**, *p* < .05). **(C):** shows confirmation of CAMKK1 overexpression in MSC transfected with CAMKK1 construct. Overexpression of CAMKK1 in MSC results in increased phosphorylation of downstream substrates including, CAMK1, CAMKIV, and AKT. GAPDH and β‐Tubulin were used as loading controls in different western blots. Abbreviations: AKT, protein kinase B; CAMK1, Ca^2+^/calmodulin‐dependent protein kinase; CAMKIV, calmodulin‐dependent protein kinase type IV; CAMKK1, calcium/calmodulim‐dependent protein kinase kinase 1; CREB, cAMP response element‐binding protein; GAPDH, glyceraldehyde 3‐phosphate dehydrogenase; pAKT, phosphorylated protein kinase B; pCAMK1, phosphorylated Ca^2+^/calmodulin‐dependent protein kinase; pCAMKIV, phosphorylated calmodulin‐dependent protein kinase type IV; pCREB, cAMP response element‐binding protein; qPCR, quatification polymerase chain reaction.

To validate the observation that expression of CAMKK1 in MSC was inversely related to Dab2 expression, we transfected MSC with Dab2:siRNA and measured CAMKK1 mRNA levels by quatification polymerase chain reaction (qPCR). We observed a ∼2.5‐fold increase in CAMKK1 mRNA compared with control cells (Fig. 3A). Overexpression of Dab2 in MSC did not significantly change CAMKK1 expression. Interestingly, TGFβ1 treatment, which has been described to reduce Dab2 levels in MSCs, increased CAMKK1 mRNA levels in MSC (Fig. 3B). Importantly, the inverse expression effect of Dab2 on CAMKK1 was also observed at the protein level (Fig. 3A, inset). These data confirm our Illumina array and indicate that CAMKK1 expression is significantly increased in cells when Dab2 is downregulated.

Confirmation of CAMKK1 overexpression in MSC transfected with CAMKK1 DNA was confirmed at the protein level by western blot (Fig. 3C). We detected changes in the phosphorylation state of proteins directly linked to CAMKK1 signaling pathway. These include increased phosphorylation of calcium/calmodulim‐dependent protein kinase 1 (CAMK1), calcium/calmodulim‐dependent protein kinase (CAMKI), cAMP (adenosine 3'5' cyclic monophosphate) response element binding protein (CREB), and AKT (Fig. 3C). These data further support the hypothesis of CAMKK1 signaling as being the main driver of positive cardiac effects observed in our animal model of AMI.

### CAMKK1 Positively Regulates Cardiac Beneficial Effects Associated with MSC Secretome

**Figure 4:**
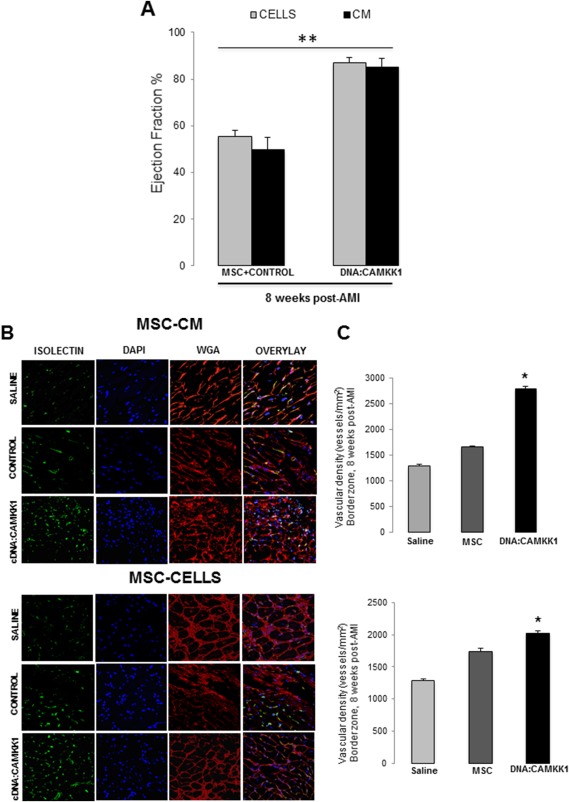
CAMKK1‐induced secretome in MSCs improves cardiac function after AMI. **(A):** Cardiac function (ejection fraction) 8 weeks after AMI, was significantly improved by MSC and CM from MSC overexpressing CAMKK1. Data are mean ± SEM (*n* = 7–8 per group) *, *p* < .01 versus control cells. **(B):** Cardiac function improvement induced by CAMKK1 overexpression in MSC correlates with increased vascular density in cardiac border zone after 8 weeks post‐AMI, as determined by confocal imaging and fluorescence intensity analysis. Endothelial cells were stained with isolectin (green), counter‐stained with wheat germ agglutinin (red), and DAPI for nuclei detection (blue). Vascular density quantification is shown in **(C)**. Data represent mean ± SEM of an *n* = 8. *, *p* < .05 versus corresponding saline treatment group. Abbreviations: AMI, acute myocardial infarction; CAMKK1, calcium/calmodulin‐dependent protein kinase kinase‐1; CM, conditioned media; DAPI, 4′,6‐diamidino‐2‐phenylindole; MSC, mesenchymal stem cells; WGA, wheat germ agglutinin.

We tested whether CAMKK1 overexpression modulates the benefits associated with the MSC secretome in cardiac function after AMI. To determine whether upregulation of CAMKK1 in MSC is sufficient to induce tissue repair, we performed studies with control and CAMKK1 overexpressing MSC, as well as CM from both culture conditions, and injected these treatments in infarcted cardiac tissue at the time of AMI. As seen in Figure 4A, MSC or CM following DNA:CAMKK1 transfection into MSC, leads to a significant improvement in cardiac function as measured by percentage of EF after 8 weeks of AMI, when compared with cells or CM from control non‐transfected cells (Fig. 4A). Histologically, CAMKK1 overexpression led to significant decreases in myocardial scar (control MSC: 50% ± 3.2% versus CAMKK1 cells: 28% ± 1.4%; *p* < .05) and vascular density (Fig. 4B, 4C).

### Direct Myocardial CAMKK1 Overexpression Improves Myocardial Response to Injury

**Figure 5:**
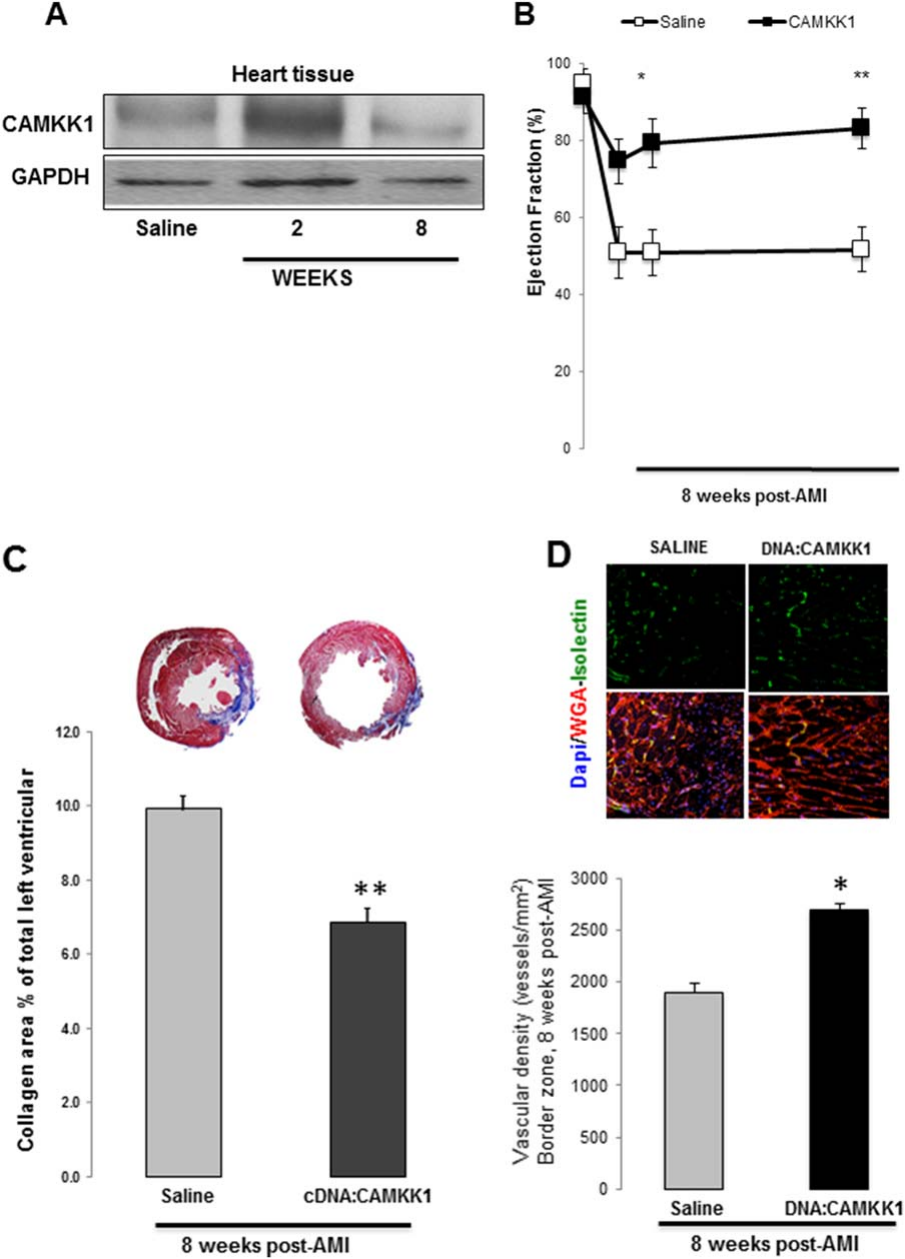
Induced intramyocardial expression of CAMKK1 improves cardiac function after AMI. Induced CAMKK1 expression in cardiac tissue was observed 2 weeks after DNA injections, returning to saline levels at week 8 **(A)**. CAMKK1:DNA intramyocardial injections at the time of AMI, resulted in improved ejection fraction measurements after 8 weeks of recovery, compared with saline injections **(B)**. Infarct size measured by Masson's trichrome stain was significantly reduced as well. Representative photomicrographs of 4 mm sections are pictured from an animal from each treatment group above each data column **(C)**. Data are presented as mean ± SEM of seven animals per group (**, *p* < .05 compared with saline control). Neovascularization as a measure of cardiac tissue recovery, was found to be significantly increased in CAMKK1:DNA injected tissue. Representative images of immuno‐fluorescent staining of isolectin and WGA from infarcted border zone of the heart **(D)**. Quantitative analysis shows blood vessel density (D, histogram). Data represent mean ± SEM, *n* = 12 per group. *, *p* < .01 versus control. Abbreviations: AMI, acute myocardial infarction; CAMKK1, calcium/calmodulin‐dependent protein kinase kinase‐1; GAPDH, glyceraldehyde‐3‐phosphate dehydrogenase; WGA, wheat germ agglutinin.

We wanted to test whether transient direct myocardial overexpression of CAMKK1 can lead to improvement in cardiac function in AMI. To test this hypothesis, we delivered, in a randomized blinded fashion, via intramyocardial injection, five doses of 100 μg of the encoding full length CAMKK1 plasmid into the infarct border zone immediately after LAD ligation using CMV as a promoter. Intramyocardial injections of CAMKK1 expression vector led to an increase in cardiac CAMKK1 expression observed 2 weeks after injection, which appear to go back to control levels (Fig. 5A). Figure 5B shows that we observed significantly increased cardiac function in those animals that received CAMKK1 expression vector through 8 weeks after AMI, as measured by percentage of EF: CAMKK1: 83.2% ± 5.4% versus Saline: 51.7% ± 5.8% (*p* < .0001, baseline: 91.3% ± 4.3%). A significant decrease in Infarct size and myocardial scar was also observed, as the percent LV area expressing collagen was reduced in hearts injected with CAMMK1 up to 35% of saline injected hearts (Fig. 5C). And, correlating with these results, we also determined by histological analysis, a significant increase in vascular density. Altogether demonstrating a clear beneficial effect of inducing local tissue expression of CAMKK1 (Fig. 5D).

## Discussion

AMI frequently impairs cardiac function, eventually leading to chronic heart failure. Cell therapy remains a potentially beneficial approach to regain cardiac function and minimize scar formation in AMI. In this regard, it has been extensively demonstrated, by us and others, that delivery of MSC in the peri‐infarct period improves cardiac function in both pre‐clinical and clinical studies 9, 14, 15. It has also been reported that the benefits of MSC engraftment might be due partially to the MSC secretome. Our group has previously demonstrated a critical role for SDF‐1 as a paracrine factor for MSC 8, 16, 17. While significant work has been done in demonstrating the benefits of the MSC secretome, there is little understanding of the molecular mechanisms that regulate the MSC secretome, or how this secretome can be manipulated for clinical gain studies 18, 19. In this work, we focused our investigation on molecular mechanisms that regulate the MSC secretome, based on our previous observations that Dab2 downregulation significantly enhances MSC function 11, 12.

Through a hypothesis driven gene array study, we identified CAMKK1 as a potential regulatory protein of the MSC secretome. Our data demonstrate that its overexpression leads to improved cardiac function in AMI through the delivery of CM from CAMKK1 overexpressing MSC, the injection of CAMKK1 overexpressing MSC or the direct overexpression of CAMKK1 in the myocardium. Furthermore, the improvement we observed with CAMKK1 overexpression correlated with an increase in vascular density and a decrease in myocardial scar. CAMKK1 is a transferase that belongs to the Ser/Thr protein kinase family, expressed primarily in central nervous system cells. Among the tissues with detectable levels are pancreas, amygdale, hypothalamus, prostate, and lung 20, 21. CAMKK1 activates CAMK1 and CAMKIV by phosphorylation of their amino acids Thr177 and Thr196 respectively and has been shown to modulate mammalian target of rapamycin complex 1 (mTORC) and 5' adenosine monophosphate‐activated protein kinase (AMPK) signaling 22, 23, 24. One of CAMKK1's principal functions is the regulation of calcium triggered signaling in cellular processes. Our data suggest that CAMKK1 functions as a modifier of MSC functional effects on tissue healing and repair and indicates that these functional effects are due to enhancement of the MSC secretome since, similar beneficial effects are seen with the delivery of CAMKK1 overexpressing MSC or CM from these cells.

## Conclusion

As a regulator of enhanced MSC secretome function, the identification of CAMKK1 will allow for a focused delineation of the intracellular pathways involved in the generation of a reparative MSC secretome, as well as detailed analysis of proteins, miRNA or exosomes of interest within the CM, that might be responsible for the benefits seen in our experimentation. Our findings suggest defining the molecular signaling linking Dab2 and CAMKK1 in future studies, could lead to the identification of important targets for possible therapeutic manipulation. Importantly, our studies are show that direct overexpression of CAMKK1 in cardiac tissue leads to enhanced tissue repair in the absence of MSC or CM from MSC. This indicates that further understanding of the down‐stream pathways activated by CAMKK1 could lead to refinement of relevant pathways involved in the generation of the reparative MSC secretome. The data presented here, further suggest that potentially any cell type can be induced to release a reparative or regenerative secretome, a concept that could significant impact the development of future therapeutics.

## Author Contributions

F.D., M.S.P., and M.E.M.: conception and design the experiments; S.P., M.K., and M.E.M.: collection and/or assembly of data; F.D., Z.H.D., and M.E.M.: data analysis and interpretation; F.D. and M.E.M.: manuscript writing; M.S.P.: financial support.

## Disclosure of Potential Conflicts of Interest

M.K., M.E.M., and M.S.P. are listed as inventors on a patent application submitted by Summa Health (Akron, OH). The other author indicated no potential conflicts of interest.
